# Synthesis and functionalization of protease-activated nanoparticles with tissue plasminogen activator peptides as targeting moiety and diagnostic tool for pancreatic cancer

**DOI:** 10.1186/s12951-016-0236-3

**Published:** 2016-12-19

**Authors:** Sophie Dobiasch, Szilard Szanyi, Aleko Kjaev, Jens Werner, Albert Strauss, Christian Weis, Lars Grenacher, Katya Kapilov-Buchman, Liron-Limor Israel, Jean-Paul Lellouche, Erica Locatelli, Mauro Comes Franchini, Jennifer Vandooren, Ghislain Opdenakker, Klaus Felix

**Affiliations:** 1 Department of Surgery, University of Heidelberg, Im Neuenheimer Feld 110, 69120 Heidelberg, Germany; 2 Department of Radiology, University of Heidelberg, Heidelberg, Germany; 3 Department of Radiation Oncology, Technische Universität München, Munich, Germany; 4 Department of General-, Visceral-, Transplantations-, Vascular- and Thorax-Surgery LMU Munich, München, Germany; 5 Nanomaterials Research Center, Institute for Nanotechnology and Advanced Materials, Bar-Ilan University, Ramat-Gan, Israel; 6 Department of Industrial Chemistry Toso Montanari, University of Bologna, Bologna, Italy; 7 Department of Microbiology and Immunology, Rega Institute for Medical Research, KU Leuven, Leuven, Belgium; 8 Diagnostik München, Diagnostic Imaging and Prevention Center, Munich, Germany

**Keywords:** Galectins, Tissue plasminogen activator, Nanotheranostics, Pancreatic cancer

## Abstract

**Background:**

Functionalized nanoparticles (NPs) are one promising tool for detecting specific molecular targets and combine molecular biology and nanotechnology aiming at modern imaging. We aimed at ligand-directed delivery with a suitable target-biomarker to detect early pancreatic ductal adenocarcinoma (PDAC). Promising targets are galectins (Gal), due to their strong expression in and on PDAC-cells and occurrence at early stages in cancer precursor lesions, but not in adjacent normal tissues.

**Results:**

Molecular probes (10-29 AA long peptides) derived from human tissue plasminogen activator (t-PA) were selected as binding partners to galectins. Affinity constants between the synthesized t-PA peptides and Gal were determined by microscale thermophoresis. The 29 AA-long t-PA-peptide-1 with a lactose-functionalized serine revealed the strongest binding properties to Gal-1 which was 25-fold higher in comparison with the native t-PA protein and showed additional strong binding to Gal-3 and Gal-4, both also over-expressed in PDAC. t-PA-peptide-1 was selected as vector moiety and linked covalently onto the surface of biodegradable iron oxide nanoparticles (NPs). In particular, CAN-doped maghemite NPs (CAN-Mag), promising as contrast agent for magnetic resonance imaging (MRI), were selected as magnetic core and coated with different biocompatible polymers, such as chitosan (CAN-Mag-Chitosan NPs) or polylactic co glycolic acid (PLGA) obtaining polymeric nanoparticles (CAN-Mag@PNPs), already approved for drug delivery applications. The binding efficacy of t-PA-vectorized NPs determined by exposure to different pancreatic cell lines was up to 90%, as assessed by flow cytometry. The in vivo targeting and imaging efficacy of the vectorized NPs were evaluated by applying murine pancreatic tumor models and assessed by 1.5 T magnetic resonance imaging (MRI). The t-PA-vectorized NPs as well as the protease-activated NPs with outer shell decoration (CAN-Mag@PNPs-PEG-REGAcp-PEG/tPA-pep1_Lac_) showed clearly detectable drop of subcutaneous and orthotopic tumor staining-intensity indicating a considerable uptake of the injected NPs. *Post mortem* NP deposition in tumors and organs was confirmed by Fe staining of histopathology tissue sections.

**Conclusions:**

The targeted NPs indicate a fast and enhanced deposition of NPs in the murine tumor models. The CAN-Mag@PNPs-PEG-REGAcp-PEG/tPA-pep1_Lac_ interlocking steps strategy of NPs delivery and deposition in pancreatic tumor is promising.

## Background

At present, no reliable method is available which allows for early diagnosis of pancreatic cancer. Clinically available imaging modalities lack the sensitivity and specificity to diagnose asymptomatic pancreatic cancer and precursor lesions. This uncertainty leads to a postponement of surgical intervention for months, during which time often incurable tumors may arise from precursor lesions or small premalignant foci [1]. Early detection is the only promising approach to significantly improve the survival of patients with pancreatic cancer. Non-invasive tools for the diagnosis and monitoring of this disease are of urgent need.

Tumor associated antigens, if highly expressed intracellularly or at the cell surface of pancreatic cancer cells, were suggested to be ideal targeting proteins for tumor-size quantification. Ideally, these antigens need to be present already at early tumor stages and not, or only in negligible amounts, in the tumor-neighboring tissues. Increasing evidence exists that galectins have important functions in several aspects of cancer biology [2] including pancreatic cancer [3]. They contribute to neoplastic transformation, tumor cell survival, angiogenesis and tumor metastasis. Galectins are present both inside and outside cells, and function both intracellularly and extracellularly. There is direct evidence that galectin-1 and galectin-3 expression is necessary for the initiation of the transformed phenotype of tumors [4]. The mechanisms by which galectins are involved in cell transformation are not yet fully understood, but both galectin-1 and galectin-3 can interact with oncogenic Ras [5–7]. Recently it was shown that galectin-1 (Gal-1) is a functional tissue plasminogen activator (tPA) -receptor participating in PDAC progression with high specificity and strong affinity and therefore provides a promising therapeutic strategy for this cancer [8]. Gal-1 was studied here because it is strongly expressed in PDAC cells and tumoral fibroblasts, and plays a crucial role in PDAC-associated desmoplasia, a main hallmark of pancreatic cancer. Expression of galectins is known to be upregulated in PDAC [9] but, more importantly, not expressed in adjacent normal tissues [9, 10]. Because the overexpression of galectins already occurs under inflammatory conditions and early stages of cancer in pancreatic cancer precursor lesions, PanINs [11] the proteins have the potential of marking cells prior to their development into cancerous lesions [12]. Therefore, galectins may be good receptors to bind and to accumulate ligand-decorated nanoparticles in pancreatic cancer cells and thus allow imaging and therapeutic tumor targeting.

Iron oxide nanoparticles received great attention due to their potential application as safe and non-toxic contrast agent for magnetic resonance imaging and have been applied in early diagnosis of cancerous lesions [13] The possibility to coat them with biodegradable and biocompatible polymers suitable for drug delivery applications is particularly appealing since it allows their administration within the body with reduction of side-effects and enhancement of tumor uptake [14]. Chitosan [15] and poly(lactic-co-glycolic acid) [16] are both able to form nanostructures and to entrap iron oxide nanoparticles. In this study we decided to test the two systems in order to evaluate their efficacy in delivery of nanoparticles.

Due to the leaky vasculature and the poor lymphatic drainage of tumors, NPs can selectively accumulate in the tumor tissue [17, 18]. They can convey additional ligands for active targeting, whereby moieties can bind with receptors overexpressed and presented on the surface of cancer cells [19]. The targeting ligands enable not only the specific nanoparticle-cancer cell interactions, but also promote internalization by receptor-mediated endocytosis.

The purposes of this study were to determine the galectins Gal-1, Gal-3 and Gal-4 known to be overexpressed in pancreatic cancers as targets and to investigate a potential ligand tPA-derived peptide as receptor/ligand binding partner. The biodegradable CAN-Mag-Chitosan-PEG and maghemite-loaded PLGA-PEG-COOH based polymeric nanoparticles (CAN-Mag@PNPs) were selected as nano-Fe-delivery systems and functionalized with the t-PA-peptide-1 with a lactose-linked serine. We further tested the concept of protease-activated NPs by addition of an outer protective PEG-shell [20]. The proteolytically cleavable outer PEG-shell allows to overcome the scavenging properties by the immune system and enables an escape from the monocyte phagocytic system (MPS). With this the CAN-Mag@PNPs-PEG-REGAcp-PEG/tPApep1_Lac_ NPs reside for extended time in the blood circulation, can more efficiently penetrate through the interstitial tumor microenvironment [21]. The presence of matrix metalloproteinases in the tumor microenvironment specifically activated the NPs binding to tumor cell surface receptors by de-shielding the NP and thus promoted the linkage of tPA-peptide1-vectorized NPs to cells by ligand-receptor (tPA-Gal) interaction. Here we show that the investigated tPA-peptide1-vectorized NPs efficiently bind and accumulate on tumor cells and stroma which allows already small tumor size detection and imaging.

## Results

### Galectin expression

**Fig. 1:**
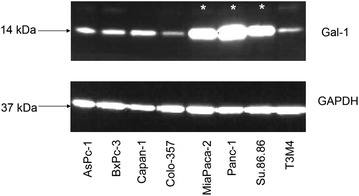
Expression of galectin-1 (Gal-1) protein in eight human PDAC cell lines. The immunoblot shows the highest Gal-1 expression in MiaPaca-2, Panc-1 and Su.86.86 cells

Tumor associated antigens, which are selectively overexpressed in PDAC cells and particularly already at early stages are ideal target proteins for early detection and tumor targeting. Based on previous work of our laboratory [9, 10] and others [3, 8, 22] we proved first the suitability of the proposed galectins as targets, and identified suitable cell lines for the experimental analysis, as well as selected ligands interacting with the galectins. For this purpose eight human pancreatic cancer cell (PaCa) lines were tested for the expression of galectin-1 at the mRNA and protein levels. Gal-1 was detected in all PaCa cells, however, the highest Gal-1 expression on mRNA and protein level were found in MiaPaca-1, Panc-1 and Su.86.86 cells as shown in Table 1 and Fig. 1.

**Table 1:** mRNA-expression of galectin-1 for the eight hPaCa cell lines

Cell line	AsPc-1	BxPC-3	Capan-1	Colo-357	MiaPaca-2	Panc-1	Su.86.86	T3M4
mRNA copies^a^	9301	1432	3838	3666	34,314	36,742	17,498	2550

^a^QRT-PCR data: adjusted to 1 × 10^4^ copies of cyclophylin B

Two cell lines Panc-1 and Su.86.86 were selected for subsequent studies.

**Fig. 2:**
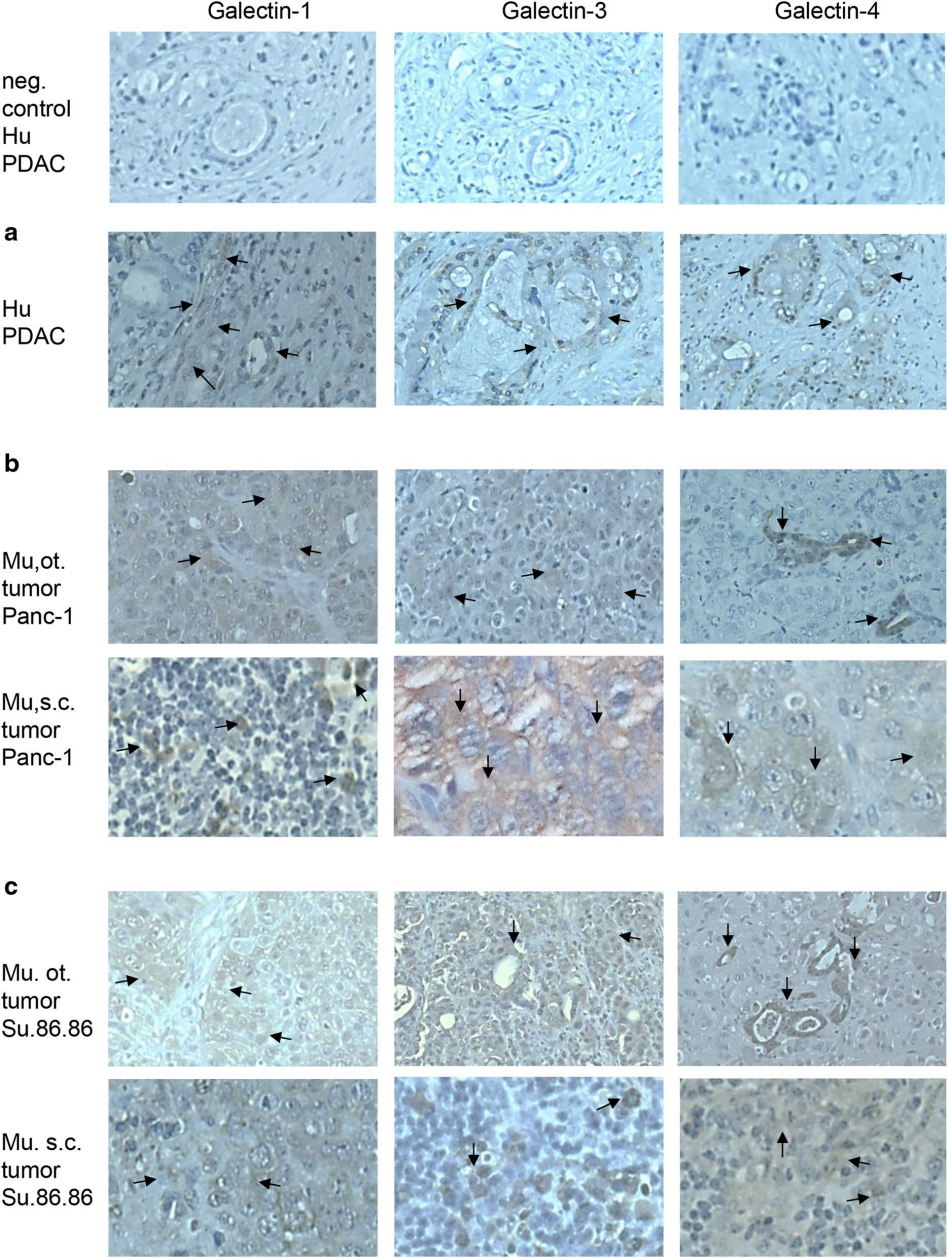
IHC confirms overexpression of galectins (Gal-1, -3, -4) in pancreatic tumors. **a** Human pancreatic ductal adenocarcinoma (Hu PDAC); **b** Murine (Mu) orthotopic (ot) and subcutaneous (s.c.) tumors of Panc-1 and **c** orthotopic and subcutaneous Su.86.86 tumors of nude mice. Some representative galectin-1, -3, -4 stained areas (cytosolic, membraneous and stroma) are indicated by *arrows*

The expression of Gal-1 was also assessed by immunohistochemistry in human PDAC-tissue. Gal-1 which is a 14 kDa protein was highly expressed intracellularly and on the cell surface in human PaCa cell lines and in PDAC-tissues. Strong staining, particularly in epithelial cells and stroma occurred likely due to Gal-1 secretion from cancer cells Fig. 2a. Additionally to human PDAC the expression of our target proteins Gal-1, Gal-3 and Gal-4 was confirmed in the below described subcutaneous and orthotopic tumor mouse models with human Panc-1 and Su.86.86 cells (Fig. 2b–d).

### t-PA-peptides: interaction studies of galectins with t-PA by thermophoresis

To investigate the binding efficiency of the short t-PA-ligands [10 to 29-AA-long peptides fluorescein or rhodamine-labelled] to galectin-1, we applied the microscale thermophoresis (MST) technology which allows quantitative analysis of protein interactions in solutions. By using MST, individual affinity constants between the different t-PA peptides and galectins (Gal-1, -3, -4) previously characterized to be over-expressed in PDAC were determined and compared to the recombinant human t-PA protein (rh t-PA) as presented in Table 2.

**Table 2:** Binding constants (K_D_) of t-PA-peptide-1 and its derivates with galectins Gal-1, Gal-3 and Gal-4

T-PA peptide	Label	Modification	Galectin	K_D_ [µM]	Fold diff.
rh t-PA protein	N-Fluo.	None = (Asn16)	Gal-1	4.92	1
Peptide-1(pep-1)	N-Fluo.	None	Gal-1	141.1	−28.7
t-PA-pep-1	C-Fluo.	None	Gal-1		
t-PA-pep-1_gal_	C-TAMRA	Ser16-*α* d-galactose	Gal-1	0.66	7.5
t-PA-pep-1_lac_	N-Fluo.	Ser16-*β* d-lactose	Gal-1	0.286	17
t-PA-pep-1_lac_	C-Fluo.	Ser16-*β* d-lactose	Gal-1	0.20	*25*
rh t-PA protein	N-Fluo.	None = (Asn16)	Gal-3	7.68	1
t-PA-pep-1	N-Fluo.	None	Gal-3	272.0	−35.4
t-PA-pep-1_gal_	C-TAMRA	Ser16b-*α* d-galactose	Gal-3	39.1	−5.1
t-PA-pep-1_lac_	C-Fluo.	Ser16b-*β* d-lactose	Gal-3	0.73	*10.4*
rh t-PA protein	N-Fluo.	none = (Asn16)	Gal-4	51.2	1
t-PA-pep-1	N-Fluo.	none	Gal-4	34.0	1.5
t-PA-pep-1_gal_	C-TAMRA	Ser16-*β* d-galactose	Gal-4	211	−4.2
t-PA-pep-1_lac_	C-Fluo.	Ser16-*β* d-lactose	Gal-4	12.7	*4.03*

The MST was performed with the tPA peptide and its variants (N- or C-terminal fluorescence-labeled and glycosylated forms) in the presence of human galectins

Italic values indicate significance of p < 0.01

Peptide-1 (AA position 137-164) in the native t-PA with additional glycosylation (lactose) and C-terminal fluorescein-labeled lysine showed the strongest binding properties to Gal-1. The binding was 25-times higher compared to that of rh t-PA. Therefore this molecule was employed as targeted moiety and for the manufacture of NPs for pancreatic tumor targeting. The 29 AA long sequence of the t-PApep-1_Lac_ is here presented: GTWSTAESGAECTNWXSSALAQKPYSGRK (X = Ser(*β*-d-lactose) and the free amino- and carboxy-terminal groups of the peptide were used as the linkage to the NPs. The side-chain of the C-terminal lysine (K) was additionally fluorescein labelled for monitoring studies.

On the basis of the affinity constants between the different t-PApep-1 and galectins (Table 2), we next assessed the cell-binding properties of NPs, functionalized with the t-PApep1_Lac_.

### Nanoparticles

**Fig. 3:**
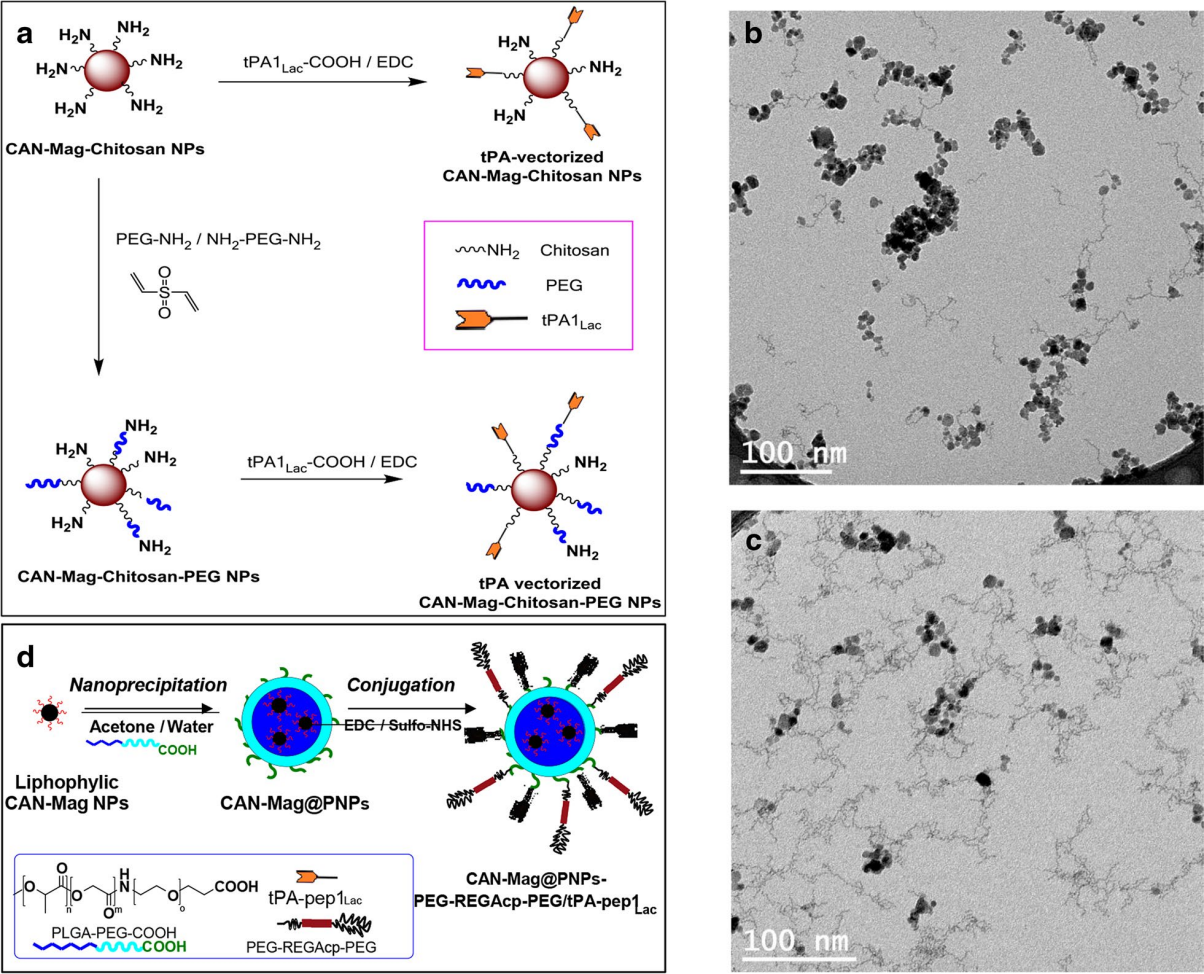
**a**–**d** Nanoparticle synthesis: **a** synthetic route to t-PA decoration of both CAN-Mag-Chitosan and CAN-Mag-Chitosan-PEG NPs. TEM microphotographs of t-PA-decorated (**b**) CAN-Mag-Chitosan and (**c**) CAN-Mag-Chitosan-PEG NPs. **d** Schematic presentation of the preparation of the final nanosystem CAN-Mag@PNPs-PEG-REGAcp-PEG/tPA-pep1_Lac_

The as-synthesized cerium cation-doped and chitosan-functionalized *γ*-Fe_2_O_3_ maghemite (CAN-Mag-Chitosan) NPs were further functionalized with PEG species of 2 and/or 5 kDa molecular weight chains. For this purpose, mono- and *bis*-amine-end-capped PEGs were mixed with chitosan-functionalized CAN-Mag NPs in the presence of divinylsulfone (DVS) that acted as a reactive linker between amine-functionalized NPs surface and PEG chain ends (Fig. 3a). To further decorate NPs with t-PA, a carboxylic acid group of the t-PA peptide was activated by EDC prior to the addition to amine-functionalized magnetic NPs. TEM images of chitosan and chitosan-PEG-modified CAN-Mag NPs are presented in Fig. 3b, c. The grafted polymers are seen as fibres connected between the NPs. The characteristics of different t-PA-vectorized CAN-Mag-Chitosan-PEG NPs are also detailed in Table 3.

**Table 3:** Characterization of nanosystems

Nanosystem	Diameter [nm]	PDI	ζ-potential [mV]	Iron [mg/ml]	Dry matter [mg/ml]	tPApep1_lac_ [µM]
CAN-Mag-Chitosan-tPApep1_Lac_ PEG 2 kDa	191.6 ± 1.5	0.44 ± 0.03	34.2	0.45	2.74	38.4
CAN-Mag-Chitosan-tPApep1_Lac_ PEG 2&5 kDa	266 ± 0.5	0.46 ± 0.03	31.3	0.45	3.8	60.1
CAN-Mag-Chitosan-tPApep1_Lac_ PEG 5 kDa	156 ± 2	0.429 ± 0.02	21.4	0.48	8.8	37.4
CAN-Mag@PNPs-tPApep1_Lac_	125.4 ± 1.8	0.133 ± 0.012	−15.4	0.636	6.0	12.9
CAN-Mag@PNPs-PEG-REGAcp-PEG	118.6 ± 1.2	0.140 ± 0.004	−18.4	0.50	5.9	–
CAN-Mag@PNPs-PEG-REGAcp-PEG/tPApep1_Lac_	114.7 ± 2.9	0.196 ± 0.004	−5.8	0.641	6.6	31.7

The DLS hydrodynamic diameters of these highly positively charged particles (ζ potential +20 to 34 mV range) are in a 150–270 nm range. Iron concentration in tested dispersions was determined by inductively coupled plasma atomic absorption spectroscopy (ICP-AES).

For the synthesis of the second nanostructure the CAN-maghemite nanoparticles were coated onto their surface with an organic ligand in order to ensure lipophilicity instead of hydrophilicity [23], then they were entrapped into polymeric nanoparticles (PNPs) made up of poly(lactic-co-glycolic)-co-polyethylene glycol copolymer [24, 25]. The as-synthesized CAN-Mag@PNPs showed good features and a negative ζ-potential due to the presence in the outer shell of deprotonated carboxylic acid groups derived from the free end of the PEG fragment. The presence of these groups allowed for the CAN-Mag@PNPs surface functionalization with tPApep-1_Lac_ peptide or PEG-REGAcp-PEG agent. After activation of carboxylic acid groups with EDC and Sulfo-NHS, the desired peptide (or both of these) was introduced in the reaction mixture so that an amidation reaction between the activated acid and a residual terminal amine function in the peptide took place. Following this procedure CAN-Mag@PNPs-tPApep1_Lac_, CAN-Mag@PNPs-PEG-REGAcp-PEG and CAN-Mag@PNPs-PEG-REGAcp-PEG/tPA-pep1_Lac_ were obtained. All the products were characterized by means of DLS for size and ζ-potential analysis, AAS for iron amount estimation, gravimetric analysis for dry matter (iron + polymer) determination and Bradford test for the determination of peptide amount conjugated on the outer shell. Results are reported in Table 3 and the entire procedure is schematically presented in Fig. 3d.

### Binding efficiency of functionalized NPs to pancreatic cancer cell lines

With the first flow cytometry experiments we analyzed the nanoparticle-core (Core CAN-maghemite (Ce-Fe_2_O_3_) and the t-PA-peptide1-lactose (t-PApep1_lac_) linked to the core. This was to determine whether the fluorescein-labelled t-PA-peptides, covalently linked to NPs, bind to Panc-1 and Su86.86 cells and if the cells are able to internalize the NPs. In a parallel set of experiments the binding of the t-PA-peptide alone (without linkage to NPs) to the pancreatic cancer cells was assessed. For the experimental set-up a concentration of t-PA peptide of 1.25 µM was applied and the cells were exposed to the NPs with and without peptides for different incubation periods of 2, 6, 12 and 24 h. Our analysis revealed that the t-PA-peptides linked to nanoparticles bind with a lower efficiency to the cells in comparison with the binding of peptides without linkage.

Further analysis showed that the physicochemical properties of the NPs and the investigated cell lines revealed strong influences on binding efficacies. It turned out that only a minor fraction (0.5%) of the negative control NP, namely CAN-Mag@PNPs, showed fluorescence. In contrast, the functionalized nanoparticle CAN-Mag@PNPs-tPApep1_Lac_ showed up to 90% fluorescence (due to the fluorescein linked to t-PApep1_Lac_). These results are in line with good linkage-stability as shown in Table 4.

**Table 4:** Flow cytometry analysis

Nanoparticle formulation	Panc-1			Su.86.86		
	2 h	12 h	24 h	2 h	12 h	24 h
t-PApep1_Lac_	2.6	87.6	86.3	25.7	45.8	36.6
CAN-Mag@PNPs -tPApep1_Lac_	37.3	85.2	74.3	Nd	Nd	Nd
CAN-Mag@PNPs-PEG-REGAcp-PEG/tPApep1_Lac_	1.4	2.2	1.8	0.3	0.5	0.9
CAN-Mag@PNPs-PEG-REGAcp-PEG/tPApep1_Lac_ + MMP9	2.2	Nd	4.1	2.9	4.3	7.8
CAN-Mag-Chitosan–tPApep1_Lac_	30.5	63.3	55.3	Nd	Nd	Nd
CAN-Mag-Chitosan-tPApep1_Lac_ PEG 2 kDa	48.3	52.3	53.5	90.1	91.7	93.7
CAN-Mag-Chitosan–tPApep1_Lac_ PEG 2&5 kDa	21.8	62.4	68.3	68.9	85.8	79.5
CAN-Mag-chitosan–tPApep-1_Lac_ PEG 5 kDa	19.5	81.9	86.7	26.3	65.2	66.0

Percentage of cells labeled with nanoparticles (NPs) after 2, 12 and 24 h incubation time. The NP binding was dependent on the NPs composition, the exposure time and cell lines tested

*Nd* not determined

The highest binding of the vectorized NPs was reached at 6–12 h exposure. Interestingly, it was observed that strong binding (even to the cell line T3M4, results not shown) occurred indicating that other binding partners (receptors) are present on these cells. We identified, additionally to Gal-1, the Gal-3 and Gal-4 as binding candidates and confirmed the flow cytometry analysis by microscale thermophoresis (MST) experiments and IHC (Table 2, Fig. 2).

As a NPs internalization criterion we used the iron uptake determination. A time-dependent exposure of NPs to cells and subcellular fractionation showed also different results depending on cell line and exposure time. Nevertheless, for non-vectorized NPs the highest Fe levels were found in the cytoskeleton fraction (45.9%) followed by the cell culture media fraction (39%), whereas for the tPA-pep1_lac_-vectorized NPs, most of the iron (up to 88%) after 6 h exposure was measured in the membraneous fractions (Table 5).

**Table 5:** Representative iron-distribution in subcellular fractions after 6 h NPs (500 µg Fe) treatment of Su.86.86 cells (2 × 10^6^ cells in 10 cm culture dish)

Analyte	No NP Fe [ng/ml]	No-vect. NP^a^ Fe [µg]	% of total Fe	Vector. NP^b^ Fe [µg]	% of total Fe
Culture media	<5	175.9	39.3	13.86	0.9
Wash buffer	<5	48.23	10.8	18.73	4.2
Cytosol	9.55	1.18	0.3	1.41	0.3
Membrane	16.8	5.07	1.1	395.6	88.1
Nuclear	<5	11.51	2.6	18.2	4.1
Cytoskeleton	5.04	205.90	45.9	11.45	2.6

^a^CAN-Mag-Chitosan PEG 2 and 5 kDa

^b^CAN-Mag-Chitosan–tPApep1_Lac_ PEG 2 and 5 kDa

### De-shielding mechanism for the NPs: MMP-targeted NPs delivery

Elevated expression of MMP-9 is associated with cancerous and inflammatory microenvironments, including PDAC [26]. We aimed to use the presence of active MMP-9 in the tumor microenvironment to potentiate targeted delivery and infiltration of NPs into the tumor tissue of pancreatic cancer patients. Therefore we evaluated (here in vitro) the presence and activity of MMP-9 with a focus on the development of protease-modifiable nanoparticles [20]. For this purpose a cleavable peptide (named REGAcp) derived from collagen II as an optimal substrate for MMP-9 was designed and a Polyethyleneglycol-REGAcp-Polyethyleneglycol (PEG-REGAcp-PEG) chain was synthesized and covalently linked to NPs.

**Fig. 4:**
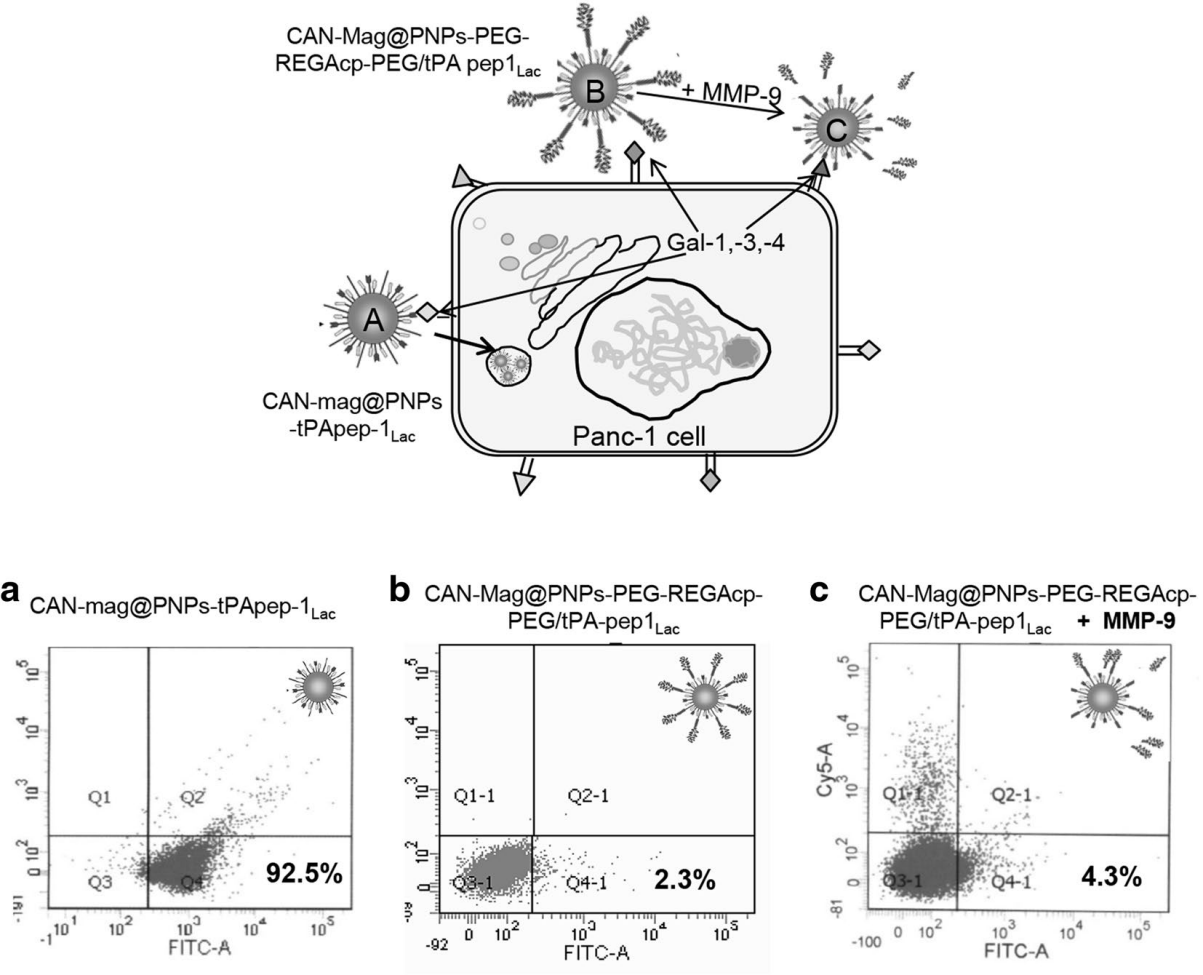
Deshielding effect. **a** The CAN-Mag@PNPs-tPApep1_Lac_ NPs bind to the receptors on the tumor cells. **b** The CAN-Mag@PNPs-PEG-REGAcp-PEG/tPA-pep1_Lac_ NPs are hindered to interact with the tumor cells due to the protective outer PEG-REGA_cp_-PEG shell. **c** The CAN-Mag@PNPs-PEG-REGAcp-PEG/tPA-pep1_Lac_ NP exposed to activated MMP-9 release the outer shell and consequently the now accessible t-PApep1_lac_ vectors are able to bind to tumor cells. Flow cytometry analysis was carried out as described in the “Methods” section. The number in the *lower right corner* depicts the percentage of NP-labeled cells

Incubation experiments with active MMP-9 indicated the de-shielding mechanism for the NPs. In this context we used MMP-9 as a tool to unmask the NPs and make these locally effective in the tumor cell vicinity. Applying the NPs CAN-Mag@PNPs-t-PApep1_Lac_, and the shielded CAN-Mag@PNPs-PEG-REGAcp-PEG/tPA-pep1_Lac_ we aimed to elucidate the binding efficiency of three modalities as presented in Fig. 4.

The results revealed that NPs with outer shell decoration (CAN-Mag@PNPs-PEG-REGAcp-PEG/tPA-pep1_Lac_) bind poorly (2.3% of the gated cells). A short (2 h) preincubation of such particles with MMP-9 showed no enhancing effect. An extended MMP-9 pre-incubation step in the presence of its activator cdMMP-3 for 24 h slightly elevated the binding (3.4–7.8%) of the NPs which was only 8.2% compared to the CAN-Mag@PNPs-tPApep1_Lac_ NPs (Table 4). The artificial simulation of the in vivo situation here demonstrated that an extended preactivation phase (deshielding step) is necessary for better binding of the particles to cells as measured by flow cytometry.

### In vivo pancreatic tumor targeting efficacy using MRI in a mouse xenograft model

We next evaluated the efficacy of NPs accumulation using xenograft models of pancreatic cancer developed by subcutaneous or orthotopic injection of Panc-1 or SU.86.86 cells under the loose skin of the flank of the hind leg or into the pancreatic tail of nude nu/nu mice. The tumor size and body weight were monitored twice a week for 30–35 days. Four to five weeks post-injection of cells, orthotopic tumors had developed to a size of approximately 300–600 mm^3^ as assessed by ultrasound imaging. On average, each tumor bearing-mouse had a body weight of 25 g (min 22.5 and 27.2 g max) and showed no adverse behavior or cachexia. Subsequently, we performed comparative tumor targeting efficacy studies by dividing animals into three groups and applying NPs with three different properties.

Using a previously established NP dose adjusted to 20 µg Fe in 150 µl NPs suspension the following regimens were administered by a single intravenous injection into the mouse tail: (i) non-targeted NPs (CAN-Mag@PNPs); (ii) tPA-pep1_lac_-decorated NPs (CAN-Mag@PNPs- tPApep-1_lac_) (iii) and tPA-pep1_lac_-decorated with outer shell REGAcp pegylated NPs (CAN-Mag@PNPs-PEG-REGAcp-PEG/tPA-pep1_Lac_). As a proof of principle MRI was applied to assess the biodistribution and to visualize the target site accumulation (subcutaneous or orthotopic tumor) of the three types of NPs. The MRI was performed at 5–8 min time intervals over a time period of 1 h.

**Fig. 5:**
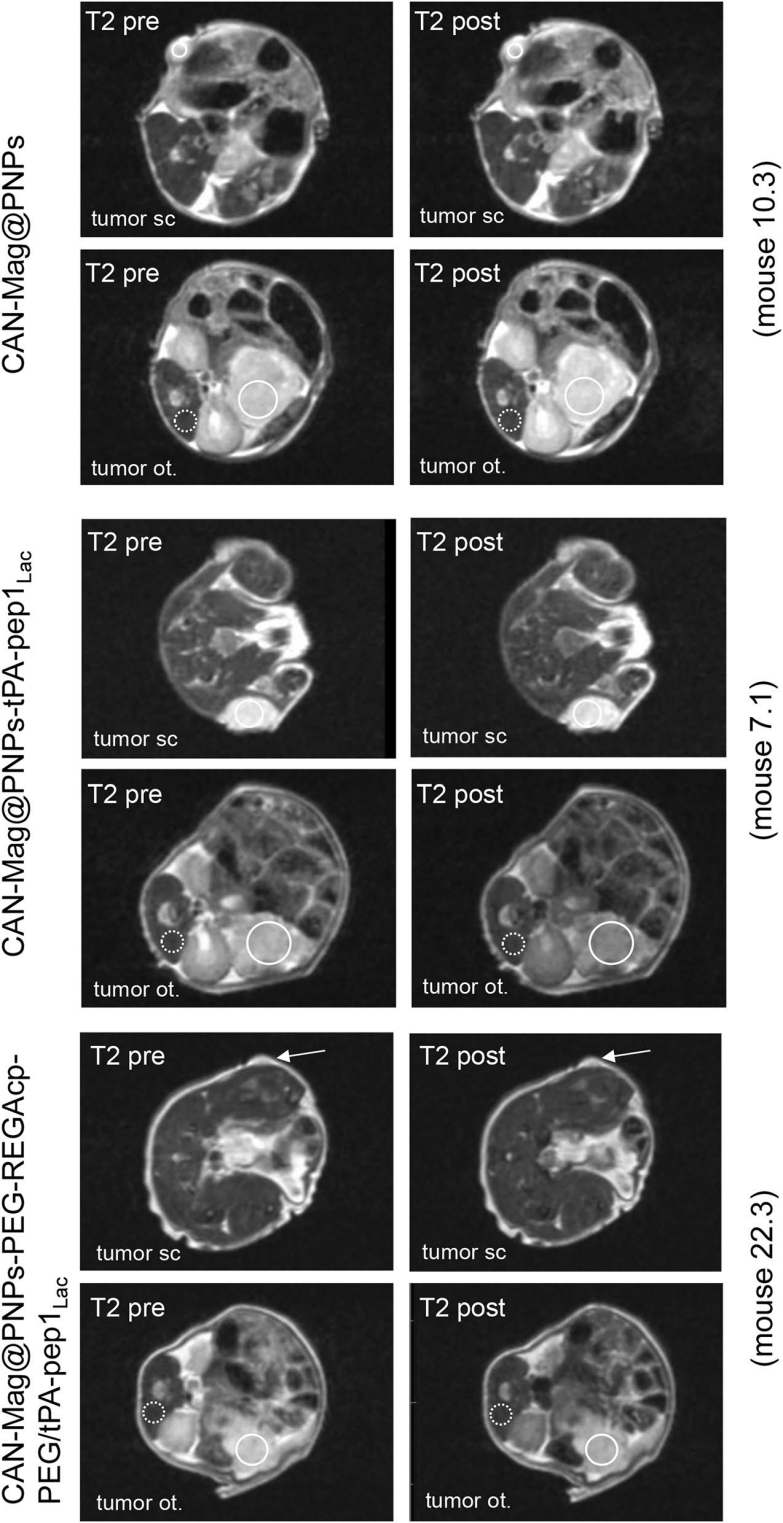
MRI: a minor portion of the injected NPs reach the tumor. Axial presentation of subcutaneous (sc) and orthotopic (ot) Panc-1 tumors T2-weighted images of a single slice before (*left*) and 5 min after injection (*right*) of CAN-Mag@PNPs (*top*); CAN-Mag@PNPs-tPApep1_Lac_ (*middle*); and CAN-Mag@PNPs-PEG-REGAcp-PEG/tPA-pep1_Lac_ NPs injection (*bottom*). The circled tumor areas represents the regions of interest (ROI) used for the analysis of signal intensity loss. The *dashed circle* areas are the muscle areas used as reference ROI. A signal drop (*darker area*) in the s.c. and ot. tumors can be seen after NP application in the T2 weighted images

The MRI results showed that a single administration of vectorized NPs (CAN-Mag@PNPs- tPApep-1_lac_) is more efficacious in tumor deposition as compared to nontargeted control NPs (CAN-Mag@PNPs). The NP-deposition was already 5 min post-injection detectable at both tumor inoculation sites but more pronounced in the orthotopic site (Fig. 5).

By comparing the ROIs voxels and their quotients between tumor and muscle of pre- and post-injection, we observed strong signal intensity (SI) quotient difference t/m of pre and post injection measured in subcutaneous and orthotopic tumors, indicating a satisfactory NP accumulation in both tumor sites in the mice. The corresponding voxel quotients of pre and post NP-injections obtained from the T2-weighted images are presented in Table 6.

**Table 6:** ROIs voxels quotients (entity/muscle) and signal intensities loss for organs of interest after administration of non-targeted and targeted NPs

Particle	Entity	SI_mean _(entity)/SI_mean_ (muscle)		Signal loss (1 − post/pre)
		Pre (t = 0 min)	Post (t = 5 min)	
CAN-Mag@PNPs	*Muscle* ^a^	232	243	−4.74
	Tumor sc.	3.11	3.21	−3.22
	Tumor ot.	3.43	3.33	2.92
	Liver	1.91	0.57	70.16
	Kidney	3.06	3.25	−6.21
CAN-Mag@PNPs-tPA-pep1_Lac_	*Muscle* ^a^	249	196	21.29
	Tumor sc.	3.52	3.21	8.81
	Tumor ot.	3.18	2.41	24.21
	Liver	1.33	0.38	71.48
	Kidney	2.54	1.69	33.46
CAN-Mag@PNPs-PEG-REGAcp-PEG/tPA-pep1_Lac_	*Muscle* ^a^	243	256	−5.36
	Tumor sc.	3.89	3.42	12.08
	Tumor ot.	4.57	3.85	15.75
	Liver	1.81	0.37	79.56
	Kidney	4.38	2.76	36.99

^a^Values are mean signal intensities not normalized to muscle

**Fig. 6:**
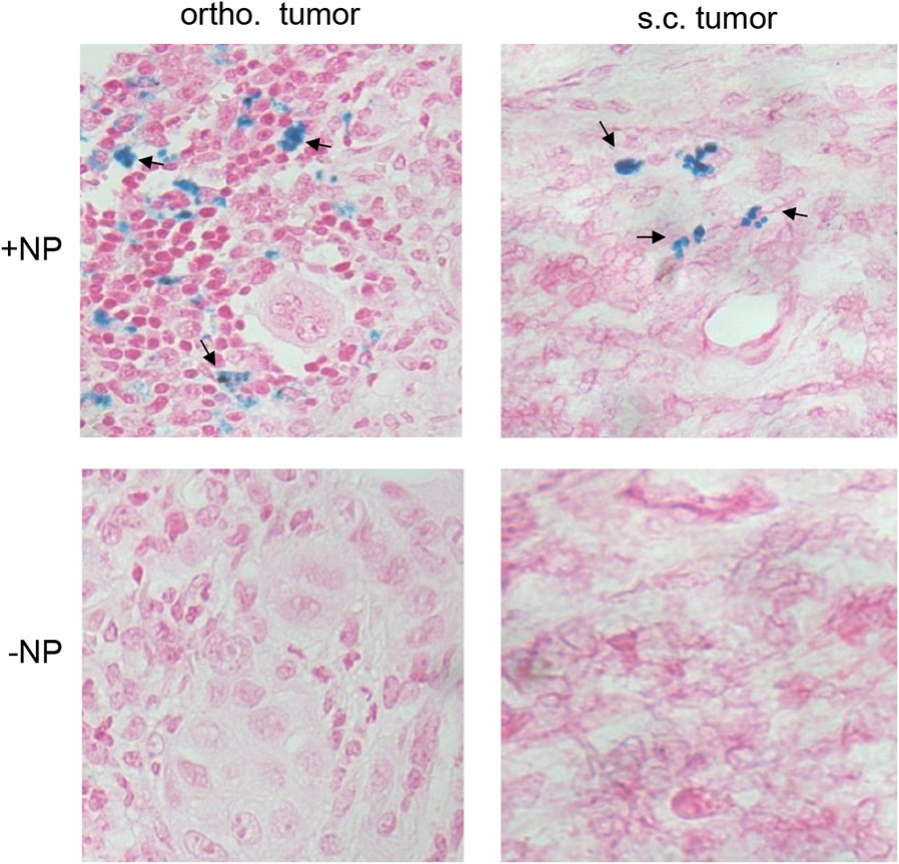
Evidence for uptake of vectorized NPs containing Fe_2_O_3_ (maghemite) in pancreatic tumors in mice shown as* Prussian blue* staining. Post MRI the animals were sacrificed and tumors and organs of interest were excised for subsequent analyses of FFPE-tissue sections for Fe-deposition (*Prussian blue*). *Top* Orthotopic (ortho.) tumor and subcutaneous (s.c.) tumor slices from a mouse ~ 1.5 h post injection of CAN-Mag@PNPs-PEG-REGAcp-PEG/tPA-pep1_Lac_ (+NP). *Bottom* Ortho. and s.c. tumor slices from an untreated control mouse (−NP). 400 × magnification; *arrows* indicate some nanoparticle deposition areas (*blue spots*)

A tumor-specific and metalloprotease-dependent NP-accumulation in the pancreatic tumor was observed by administration of vectorized NPs with outer shell REGAcp-PEGylated NPs (CAN-Mag@PNPs-PEG-REGAcp-PEG/tPA-pep1_Lac_) shown in the bottom part of Fig. 5b. Here the measurable NP-deposition occurred in some mice with delay particularly in the orthotopic tumors, however, 50–60 min after NP-injection a considerable retention in tumor was observed indicated as an intensity signal drop. One reason for this enhanced efficacy may be that the targeted particles are designed to bind to the galectins Gal-1, Gal-3, Gal-4 proteins on pancreatic cancer cells or galectin-rich tumor-stroma, thus possibly delaying clearance from the site of the tumor. Additionally, in the tumor microenvironment, elevated and active MMP-9 have first to digest the outer protective shell of the NPs and make the tPApep-1_lac_ ligands more accessible to the galectins. This proteolytic process may also be an explanation for the delayed deposition. Likely, the targeted NPs are also internalized after binding to cells, as demonstrated by previous Fe—uptake in vitro NPs exposure in cell culture. To confirm the MRI data we performed histological staining for iron (Prussian blue stainings) of the excised tumors and the tissue and the slides were evaluated (Fig. 6). Indeed, we corroborated enhanced iron deposition in NP-treated tumor samples.

## Discussion

The use of targeted drug delivery systems can significantly improve the therapeutic efficiency of small molecule chemotherapies by enhancing accumulation of the drugs in the tumor [20, 27–31]. As a positive follow-up of our previous report [32], we here explored the potential of vectorized biodegradable nanoparticles with an outer protective PEG shell cleavable by MMPs. Through this removable coating the NPs were less filtered out by the monocyte phagocytic system and resided for extended time in the blood circulation. An effective ligand directed delivery was achieved through the presence of MMP-9 in the tumor microenvironment which specifically enhanced the NPs binding to tumor cell surface receptors by de- shielding the NP and thus promoting the linkage of NPs to cells by ligand-receptor interaction and improving tumor-to-normal NP-tissue deposition ratios.

In this study we used tissue plasminogen activator protein-derived peptides as ligands and the galectins as targets on pancreatic tumor cells. The binding properties of the selected t-PA peptide-1 to galectin-1 (Gal-1) differed and depended on several factors such as C- or N- terminal fluorescence label and linkage of additional sugars as Gal-interacting molecules. Additionally to Gal-1, also Gal-3 and Gal-4 revealed to be good binding partners for the t-PA peptide-1. Applying IHC we confirmed the overexpression of galectins (Gal-1, -3, -4) not only in human pancreatic cancer tissue sections but also in here investigated subcutaneous and orthotopic murine pancreatic tumor models.

As assessed by flow cytometry analysis, there was a difference in binding efficiency of the tPA peptides to galectins depending on whether they were free or linked to nanoparticles. In general, free peptides bound to much higher degree to cells in comparison with t-PA-peptides decorated on NPs. Several of the selected t-PA peptides revealed stronger binding to Gal-1 in comparison with the native human t-PA protein. Enhancement of the binding to galectins Gal-1, Gal -3, Gal -4 by modifications of t-PA peptides was observed. Nevertheless, the t-PA peptide-1 outperformed others, particularly with a C-terminal linkage of fluorescein. Additional O-linked glycosylation of serine (S16) with lactose enhanced the binding up to 25-fold and, therefore, this glycopeptide was selected as the best binding partner (ligand) for Gal-1, also for Gal-3 and Gal-4. Flow cytometry analysis was successfully used to test NPs stability and active targeting efficiency to pancreatic cancer cells. Depending on the physicochemical properties of the investigated NPs, and of exposure times, as well as used cell lines, the binding to cells varied between 35 and 90%. NP-modification, such as the cleavable outer PEG-shell, showed approximately threefold less interactions for PEG-REGAcp-PEGylated NPs, probably due to shielding the tPA- Gal interaction. In vitro deshielding by addition of preactivated MMP-9 enhanced the binding, however, the optimal in vitro conditions were not reached.

In our in vivo experiments, a good tolerance of the investigated bioactive nanoparticles (with and without PEG-REGAcp- PEGylation) after i.v. injection in nude mice was observed. Our tests of up to 48 h post injection showed no changes in physical conditions and behaviour. The delivery of NPs to solid tumors is known to be challenging due to (i) major uptake by the macrophage phagocytosing system (MPS), for instance in the liver, and (ii) the poor penetration of the relatively large nanoparticles through the interstitial tumor microenvironment [33]. Here we investigated NPs with PEG formulations that ranged in sizes between 110 and 130 nm. PEG is frequently used as coating material for modifying the surface of NPs. PEG molecules form a protective hydrophilic layer with a negative zeta potential that helps to avoid recognition by the immune system, thereby reducing the rate of uptake of NPs by the MPS and extending their dwell time in the blood circulation [34]. Another important advantage of such PEG outer-shell decorated NPs is their accumulation in tumors due to an property termed the “enhanced permeability and retention effect” (EPR) [35]. The EPR depends upon irregular fenestration due to irregular lining of endothelial cells during the tumor neovascularization process. The irregular cell organization and leaky nature of the tumor vasculature allows a selective retention of even larger particles (up to 500 nm) due to their proclivity to leak out of the blood vessel more readily than they can permeate back into the circulation. The EPR effect, initially described over two decades ago, is already successfully exploited in the nanomedicine field and led to re-engineering of conventional chemotherapeutics. Paclitaxel is such an example: by linking the drug taxol or the synthetic derivates to nanocore systems such as human serum albumin results in Abraxane, a nanotechnology recently also applied for the treatment of pancreatic cancer [36]. However, many of these re-formulations are passive non-targeting deliverers. Now more effort and focus is towards developing NP-formulations with properties possessing targeting carriers where the optimal binding activity is peritumoral or near the tumor cell due to e.g. proteolytic activity in the tumor microenvironment and by that enhancing NPs accumulation in tumor tissue and improving the tumor to normal tissue ratios [20].

For this purpose, we developed and tested such NPs (CAN-Mag@PNPs-PEG-REGAcp-PEG/tPA-pep1_Lac_). Here, as a proof of principle, we used a vectorized nanodiagnostic without a warhead (therapeutic cargo of anticancer drug) and were able to demonstrate that such particles are more efficiently deposed in pancreatic s.c. tumor models in comparison with NPs without outer shell [32]. Additionally, applying MRI we could demonstrate that a more efficient NP deposition was observed in orthotopic tumors in comparison with the subcutaneous tumors. The orthotopic tumor model offers advantages over subcutaneous, because it can reflect the primary tumor microenvironment affecting blood supply, neovascularization, peritumoral inflammation and tumor cell invasion. Indeed, the investigated NPs ended up in the tumor, as demonstrated by the Fe-deposition in tissues of pancreatic tumor models by Prussian blue indicative for effective targeting. The higher NP accumulation in the orthotopic tumor model was likely due to the better tumor vascularization, whereas in our subcutaneous model frequently a necrotic area in the tumor center was observed. That the site of cancer cell inoculation (here subcutaneous versus orthotopical) influences tumor vascular permeability was previously reported [37].

Additionally as above mentioned, the permeability of the tumor vasculature is the consequence of an imbalance between the formation and maturation of new blood vessels. This imbalance leads to a discontinuous joint between endothelial cells and results in inter-endothelial cell gaps forming a so called fenestrated endothelium or fenestrations facilitating the NP extravasation into the tumor stroma. Furthermore, by the liberation of the outer protective NP shield by MMP-9 and negative zeta-potential, the NPs were able to reside for a more prolonged time in the tumor cell vicinity, being retained by formation of t-PApep1_lac_-Gal complexes and probably not eliminated due to a compromised lymphatic drainage known to be impaired in tumors.

## Conclusions

The in vivo functionality of the investigated NPs depended on tumor cell-type and mouse model used, and on tumor inoculation area. An effective ligand directed delivery was achieved through the presence of proteases in tumor microenvironment which specifically enhanced the NPs binding to tumor cell surface receptors by de- shielding the NP and thus promoting the linkage of NPs to cells by ligand-receptor interaction and improving tumor-to-normal NP-tissue deposition ratios. The preferential accumulation of NPs in the tumor vicinity raises the imaging sensitivity in MRI thus allowing imaging of already very small tumors (<0.5 cm^3^) of PDAC. The overlapping results (MRI and Fe-staining) with targeted NPs indicate a fast and enhanced deposition of NPs in the tumor. While further studies addressing the biodistribution and kinetics are needed to demonstrate the CAN-Mag@PNPs-PEG-REGAcp-PEG/tPA-pep1_Lac_ NPs real potential, this interlocking steps strategy of NPs delivery and deposition in pancreatic tumor is promising.

## Methods

### Cell lines

Human pancreatic carcinoma cell lines Aspc1, BxPc-3, Capan-1, MiaPaca-2, Panc-1, and Su.86.86 were obtained from American Type Culture Collection (Manassas, VA, USA). COLO-357 and T3M4 were a gift from R. Metzgar (Duke University, Durham, NC, USA). Cells were cultured in RPMI-1640 medium supplemented with 10% fetal bovine serum (FBS), 100 U/ml penicillin, and 100 µg/ml streptomycin (Invitrogen GmbH, Karlsruhe, Germany) at 37 °C in a humidified 5% CO_2_ atmosphere.

### mRNA, cDNA, QRT-PCR

All reagents and equipment for mRNA and cDNA preparation were purchased from Roche Applied Science, Mannheim, Germany. mRNA was prepared by automated isolation using the MagNA Pure LC instruments. RNA was reverse transcribed into cDNA with the use of the 1st Strand cDNA Synthesis Kit for RT-PCR (AMV) according to manufacturer’s instructions. QRT-PCR was performed with the Light Cycler Fast Start DNA SYBR Green kit. The number of specific transcripts was normalized to the housekeeping gene cyclophilin B (CPB) and presented as copies/10,000 copies of CPB.

### Protein isolation, SDS-PAGE and Western Blot

Proteins from 80 to 90% confluent cells cultures were isolated using the Pierce RIPA cell lysis buffer (ThermoScientific, Germany) and the protein concentration of the lysates was assessed by Pierce BCA Protein Assay (ThermoScientific). Protein samples were heated for 10 min at 95 °C and separated by SDS–polyacrylamide gel (10%) electrophoresis. After blotting to a nitrocellulose transfer membrane (Whatman, Dassel, Germany), a rabbit monoclonal antibody to galectin-1 (Acris 1:2000), or to control for equal loading mouse monoclonal antibody to GAPDH (BD Pharmingen, Heidelberg, Germany; 1:2000) diluted in 5% BSA, 1× TBS and 0.1% sodium azide (Calbiochem/Merck, Darmstadt, Germany) was added (at 4 °C overnight). After washing, membranes were incubated with a goat anti-rabbit IgG POX, respectively, goat anti-mouse IgG POX (BD Biosciences, Heidelberg, Germany) as the secondary antibody (room temperature for 30 min). For detection, Amersham ECL plus Western Blotting Detection System (GE Healthcare, Munich, Germany) was used.

### Analysis of biopsies

For IHC detection paraffin-embedded tissue sections (4 μm) were analyzed using the method previously described with some modifications [38]. Briefly, prior to antibody incubation, heat pretreatment in an antigen retrieval solution (DAKO Cytomation, Hamburg, Germany; using citrate buffer (pH 6.1) was performed. Primary antibodies included mouse monoclonal antibodies to Gal-1 (diluted 1:200), mAb Gal-3 (diluted 1:1000) and mAb Gal-4 (diluted 1:250) all from Acris (Acris Antibodies, Inc., San Diego, CA, USA) and were diluted in Antibody Diluent (DAKO North America, Inc., Carpinteria, CA, USA). EnVision + System-HRP, labeled polymer anti-mouse (Dako North America, Inc.) was used as secondary antibody. Isotype-specific negative controls to the primary antibodies (mouse IgG1 and mouse IgG2a, both DAKO Cytomation, Hamburg) were performed to detect the specificity of the antibodies.

In order to assess the iron oxide-loaded NPs deposition in pancreatic tumor tissue and liver in mice treated with NPs a *post mortem* qualitative approach was applied using the Prussian blue staining on histopathological formalin-fixed and paraffin-embedded (FFPE) tissue sections. Briefly the deparaffinized tissue sections were first incubated in a 10% solution of potassium ferrocyanide K_4_[Fe(CN)_6_] × 3H_2_O for 5 min. Subsequently the slides were incubated in a freshly prepared 1:2 mixture of 20% potassium ferrocyanide and 4% HCl allowing to react with the maghemite generating the dark blue complex Fe_4_[Fe(CN)_6_]_3_ × H_2_O so called Prussian blue. The slides were washed ×3 in distilled water and subsequently for 5 min counterstained with a 0.1% nuclear fast red-aluminium sulfate solution (Roth, Karlsruhe, Germany).

### t-PA peptides and interaction studies with galectins

Tissue plasminogen activator (t-PA) which was already previously identified as a potential interacting ligand for Gal-1 was selected as vector moiety [8]. However, due to the limited t-PA-protein half-life time, we selected from the native t-PA protein four molecular probes (9–28 amino acids (AA) long peptides with areas carrying N-linked glycosylation sides. The peptides derived from the human t-PA and selected as binding partners to Gal-1 were further designed (Tables 7, 8) and submitted for commercial larger scale synthesis to Peptide Synthesis Laboratories (PSL GmbH, Heidelberg, Germany).

**Table 7:** Selected human tissue plasminogen activator (t-PA)-derived peptides used for the galectin interaction studies

Peptide #	AA (n)	Amino acid sequence	Position in the t-PA^a^-protein	MW [Da]
1	28	GTWSTAESGAECTNWNSSALAQKPYSGR	137–164	2959.3
2	27	CFNGGTCQQALYFSDFVCQCPEGFAGK	91–118	2920.2
3	27	YSSEFCSTPACSEGNSDCYFGNGSAYR	198–224	2902.1
4	9	CTSQHLLNR	476–484	1071.3

^a^According position in the human tPA precursor (http://www.uniprot.org/uniprot/P00750)

**Table 8:** t-PA-peptide-1 and its modifications used for the galectins Gal-1, -3, -4 interactions studies

Peptide 1	AA(n)	Amino acid sequence	MW [Da]
N	28	Fluo-GTWSTAESGAECTNW**N**SSALAQKPYSGR-COOH	3317.4
X: S(*α*-galct.)	29	H_2_N-GTWSTAESGAECTNW**X**SSALAQKPYSGRK-TAMRA	3675.6
X: S(*ß* d-lac.)	29	H_2_N-GTWSTAESGAECTNW**X**SSALAQKPYSGRK-Fluo	3742.6

In order to investigate the binding properties of the modified t-PA-peptides [10-29 AA-long fluorescence-labeled peptides] as ligands to galectins, the microscale thermophoresis (MST) (Monolith NT.115 MST instrument, NanoTemper Technologies GmbH, Munich Germany) technology was employed [39]. MST allows a quantitative analysis of protein interactions in solution and is based on the directed motion of molecules in temperature gradients generated locally by an infrared laser, whereas the molecular mobility in the temperature gradient is analyzed via fluorescence. MST is highly sensitive to all types of binding-induced changes of molecular properties like protein size, charge, hydration shell or conformation. The binding modes such as dimerization, cooperativity, and competition are quantifiable and adaptable to diverse requirements of different biomolecules. Therefore, MST turned out as an ideal method for the interaction studies of t-PA and t-PA-derived peptides with galectins.

The prescreening entailed selecting t-PA peptide1 (AA position 137-164) in the native t-PA for the manufacture of NPs for PDAC tumor targeting, which is described here. The 29 AA long sequence of the t-PApep1_Lac_ is GTWSTAESGAECTNWXSSALAQKPYSGRK (where X stands for Ser(*β*-d-lactose). By using the free amino- and carboxyl-terminal groups of the peptide, the linkage with NPs can be performed. The side chain of the C-terminal lysine (K) is additionally fluorescein-linked for monitoring studies.

### Synthesis and characterization of t-PA-vectorized nanoparticles

Most of the specific chemicals and reagents (analytical grade and/or highest purity level) used for manufacturing and surface modification of *γ*-Fe_2_O_3_ maghemite (CAN-Mag) NPs i.e., FeCl_3_·6H_2_O, FeCl_2_·4H_2_O, NH_4_OH (28–30%), chitosan (50–190 kDa), divinylsulfone (DVS), 1-ethyl-3-(3-dimethylaminopropyl) carbodiimide (EDC) have been purchased from Sigma-Aldrich (Israel) and used without any further purification. Monoelectronic CAN [Ce^IV^(NH_4_)_2_(NO_3_)_6_] oxidant was purchased from Acros Organics. Reactive functional PEGs (methoxy-PEG amine 2 and 5 kDa, PEG diamine 2 and 5 kDa and methoxy-PEG vinylsulfone 5 kDa) were purchased from JenKem Technology USA Inc.

tPA peptide (MW 3742.6) with a full name Pep1N16(S(β-d-Lact)), with the amino-acid sequence GTWSTAESGAECTNWXSSALAQKPYSGRK (X = Ser-β-d-Lact), and with fluorescein on the K29 side-chain and with a free C-terminal group was purchased from PSL GmbH, Heidelberg, Germany. Amicon^®^ Ultra 15 ml 100 kDa centrifugal tubes were purchased from Merck Millipore.

#### Transmission electron microscopy

Both sizes and shapes of functional nanoparticles were obtained by using a transmission microscopy (FEI Tecnai Spirit Bio-Twin, Oregon, USA) equipped with a CCD 1 × 1 k camera (Gatan). Samples for TEM imaging were prepared by placing a drop of diluted H_2_O dispersion (200–250 μg/ml) onto a 400-mesh copper TEM grid (400C-FC, Electron Microscopy Sciences, Hatfield, PA, USA) and then drying in a vacuum chamber at ambient temperature.

### Nanocarrier fabrication—experimental procedure towards starting near neutral magnetite (Fe_3_O_4_) nanoparticles

This experimental procedure has been already published and described previously [15]. A solution of FeCl_3_·6H_2_O (240.0 mg, 0.9 mmol) dissolved in deoxygenated milliQ purified H_2_O (4.5 ml) was mixed with an aqueous solution of FeCl_2_·4H_2_O (97.5 mg, 0.45 mmol, 4.5 ml H_2_O). This solution was kept under N_2_ and ultrasonicated (bath sonicator) for 5–10 min at room temperature. Then, a concentrated 24 wt% aqueous NH_4_OH (0.75 ml) was introduced in one shot, resulting in an immediate black precipitation of magnetite (Fe_3_O_4_) NPs. Ultrasonication was then continued for 10 additional minutes. Resulting Fe_3_O_4_ NPs were transferred into a glass bottle (100 ml), magnetically decanted (using a strong external magnet), and washed with ddH_2_O (3 × 40 ml) until neutrality. Then, brilliant black free flowing magnetite (Fe_3_O_4_) NPs were stored as a 30 ml NPs suspension in ddH_2_O for 2 h at room temperature before any further processing/surface engineering.

### Nanocarrier fabrication—experimental procedure towards functional CAN-Mag-Chitosan NPs (chitosan decoration by injection method)

The former aqueous magnetite NPs suspension (60 ml) was magnetically decanted to separate the magnetite (Fe_3_O_4_) NPs from its aqueous storage phase. Ceric ammonium nitrate (CAN, (NH_4_)_2_Ce(IV)(NO_3_)_6_, 300.0 mg, 0.547 mmol) dissolved in 12.0 ml MeCOMe was then introduced into decanted magnetite NPs followed by the addition of degassed milliQ purified H_2_O (12.0 ml). The corresponding medium mixture was ultrasonicated using a high-power sonicator (Sonics^®^, Vibra cell, 750 Watt, power modulator set-up at 25%) equipped with a titanium horn (45 min, 0 °C) under an inert argon atmosphere. Then, a slightly acidic chitosan solution (7.5 ml, 8.0 mg/ml) was added while ultrasonication was maintained for 15 additional minutes under these same conditions. Thus, the resulting highly stabilized hydrophilic CAN_-_Mag-Chitosan NPs were cleaned [washing with ddH_2_O (3 × 10 ml) using an Amicon^®^ Ultra-15 centrifugal filter devices (100 K) processed at 4000 rpm during 5–6 min (rt, 18 °C) and re-dispersed in ddH_2_O (10 ml).

### Nanocarrier fabrication—experimental procedure for PEG attachment onto CAN-Mag-Chitosan NPs

The previously prepared and purified CAN-Mag-Chitosan NPs were analysed for weight concentration determination by freeze-drying a known volume (0.5 ml) of the NPs suspension in 2 ml polyethylene tubes and by weighing masses *before* and *after* sample loading. PEG-NH_2_ (2 or 5 kDa, 36.0 mg) and NH_2_-PEG-NH_2_ (2 or 5 kDa, 18.0 mg) were then added to CAN-Mag-NPs (10.0 mg, 3.0 ml), followed by the addition of divinylsulfone (DVS, 2.0 μl) that served as a bifunctional Michäel-reactive linker for both NPs surface and PEG species amines. Therefore, the reaction medium was shaken overnight at room temperature (RT). The resulting PEG-modified NPs were then washed/cleaned using dialysis membranes (12–14 kDa).

### Nanocarrier fabrication—experimental procedure for t-PA decoration of surface-modified CAN-Mag NPs

In the next NPs functionalization step, 1-ethyl-3-(3-dimethylaminopropyl) carbodiimide (EDC, 0.77 mg, 4.0 mmol, 0.5 ml) was added to the tPA peptide solution (0.5 mg, 0.5 ml). After 10 min reaction (RT) for COOH chemical group activation, the tPA/EDC solution was added to filtered (nylon 0.22 μm) surface-modified CAN-Mag-Chitosan or CAN-Mag-Chitosan-PEG NPs (10.0 mg, 3.0 ml) and the reaction medium was shaken overnight at RT. For resulting NPs purification step completion, the resulting functional t-PA-decorated NPs have been washed by processing in Amicon^®^ Ultra-15 centrifugal filter tubes (2 × 10 ml).

### Nanocarrier fabrication—experimental procedure towards functional CAN-Mag@PNPs

Poly(d,l-lactide-*co*-glycolide) (50/50) with carboxylic acid end group (PLGA-COOH, inherent viscosity 0.12 dl/g, MW ~7 kDa) has been purchased from Lakeshore Biomaterials (Birmingham, AL, USA). Polyethylene glycol with both amino and carboxylic acid end groups (NH_2_-PEG-COOH/NH_2_, MW ~3 kDa) has been purchased from Rapp Polymere GmbH (Tübingen, Germany). PEG_REGAcp_PEG substrate [PEG3400-Gly-Gly-Gly-Glu-Arg-Gly-Pro-Pro-Gly-Pro-Gln-Gly-Ala-Arg-Gly-Phe-HyP-Gly-Thr-Pro-Gly-Leu-PEG5000-NH_2_ (10.2 kDa, HyP = (2S,4R)-4-hydroxy Pyrrolidine-2-carboxylic acid) was purchased from Anaspec Inc. (Fremont, California, USA).

DLS measurements were performed on a Malvern Zetasizernano-S system with a 532 nm laser beam. ζ potential measurements were conducted using DTS1060C-Clear disposable zeta cells at 25 °C. SpectraAA 100 Varian was used for atomic absorption spectroscopy (AAS) analyses. Intradermal air pouch leucocytosis was applied as an in vivo the quality control for biocompatibility, toxicity and inflammatory response and the results of the core NPs were previously reported [40].

### Nanocarrier fabrication—experimental procedure towards CAN-Mag@PNPs conjugated with t-PA1_Lac_ and/or PEG-REGAcp-PEG species

The maghemite-loaded PLGA-PEG-COOH based polymeric nanoparticles (CAN-Mag@PNPs) were prepared according to a procedure already reported by us [24]. Brefly, the original CAN-maghemite nanoparticles in water were added to an ethanol solution containing the organic ligand ethyl 12-([3,4-dihydroxyphenethyl]amino)-12-oxododecanoate. The mixture was kept in an ultrasound bath for 1 h, then left to react overnight at room temperature under mechanical stirring. Afterward, the solution was decanted magnetically and washed with ethanol before redispersion of the lipophilic obtained nanoparticles in acetone.

To 10 ml of the acetone solution a total of 100 mg of the copolymer PLGA-b-PEG-COOH (10 kDa) was added. This organic phase was mixed to 100 ml of water under vigorous stirring, maintaining the water/organic ratio of 10/1 with constant removal of the resulting solution. The final mixture was kept for 30 min under vigorous stirring. The residual organic solvent was evaporated under reduced pressure. The solution of the obtained CAN-Mag@PNPs was concentrated to a volume of 10 ml using a tangential flow filter (Pellicon XL filter device, Biomax membrane with 500.000 NMWL; Millipore Corporation) following by filtration using a syringe filters SterivexTM-GP of polyethersulfone (0.22 μm, Millipore Corporation).

For the conjugation of t-PA1_Lac_ peptide and/or PEG-REGAcp-PEG the following procedure was adopted: a water solution (10 ml) of CAN-Mag@PNPs containing approximately 4 µmol of COOH functions was mixed with 10 ml of PBS 0.01 M solution. 1-Ethyl-3-(3-dimethylaminopropyl)carbodiimide hydrochloride (EDC·HCl, 4 µmol, 0.77 mg) and *N*-hydroxysulfosuccinimide (Sulfo-NHS, 4 µmol, 0.86 mg) were added and the solution was shaken for 1 h at room temperature. Then tPA1_Lac_ peptide (0.129 µmol, 0.5 mg) and/or PEG-REGAcp-PEG (0.06 µmol, 0.64 mg) were added to the reaction mixture, which was allowed to shake for an additional 24 h. The resulting nanosystem was washed with PBS 0.01 M (3 × 10 ml) using centrifugal (3000 rpm, 10 min each cycle) filter devices (Amicon Ultra, Ultracel membrane with 100.000 NMWL, Millipore, USA) and finally filtrated through Sterivex filter (0.22 µm). The final volume was adjusted to 5 ml.

### Flow cytometry

Flow cytometry (fluorescent-activated cell sorting/FACS) analysis was used to determine the binding of fluorescein-labeled t-PA-pep1_Lac_ and t-PApep1-vectorized nanoparticles (CAN-Mag@PNPs-tPApep1_Lac_ and CAN-Mag@PNPs-PEG-REGAcp-PEG/tPA-pep1_Lac_) on the surfaces of Panc-1 and SU.86.86 cells. Additional FACS analyses were performed to monitor concentration and time-dependent binding efficiency and changes caused by addition of competitors (galactose, lactose) or in the presence of active MMP-9, which cleaves the REGAcp sequence. Deshielding of CAN-Mag@PNPs-PEG-REGAcp-PEG/tPA-pep1_Lac_ was carried out by pre-activated MMP-9 (20 µM proMMP-9 and 200 nM of catalytic domain cdMMP-3 in 100 mM Tris, 100 mM NaCl, 10 mM CaCl_2_, pH 7.4 for 2 h at 37 °C). The active MMP-9 (50 µl, 10 µM) was incubated in the presence of 450 µl NPs and subsequently added to cells. Nanoparticle-exposed cell monolayers (of 2 × 10^5^ cells/2 ml medium in 6 well plates), with or without treatment, were harvested 2, 12, 24 h post-exposure using 1 mM EDTA–phosphate-buffered saline (PBS) detachment buffer, (Miltenyi GmbH, Bergisch Gladbach, Germany), washed 3 times in PBS, incubated for 45 min on ice and subsequently subjected to analysis in a BD™ LSR II flow cytometer and Software Program: BD FACSDiva 7.0 (Becton–Dickinson,)

### Iron uptake

For the determination of NP binding and uptake in Panc-1 and Su.86.86 cells the culture medium of a T-75 flask at 80–90% cell culture confluency cells (3–4 × 10^6^ cells) was replaced by PBS and the cells were exposed for 2, 4 and 6 h to NP with an iron content of 500–600 µg Fe/T75 flask. Two sets of NPs were used: CAN-Mag-Chitosan (*non*-*targeted*) and the corresponding CAN-Mag-Chitosan-t-PApep1_Lac_ (*targeted*). After NP-exposure the cell culture supernatants were collected and the adherent cells were processed in a wash buffer and the subcellular fractions were performed using the ProteoExtract™-kit (Calbiochem/Merck, Darmstadt, Germany) for the isolation of subcellular compartments (membrane, cytoplasm, nucleus, cytoskeleton), according to the manufacturer’s recommendation. The collected cell culture supernatants and fractions were transferred into glass flasks, dried for 72 h at 80 °C and subsequently processed for Fe-determination with 2 ml 65% HNO_3_ and 250 µl 30% H_2_O_2_. The inorganic residues were resuspended in 1 ml 2% HNO_3_ and the Fe-content was determined by inductively coupled plasma-optical emission spectrometry using Agilent ICP-OES 720 (Agilent Technologies, Santa Clara, CA).

### Tumor mouse models

For the subcutaneous (s.c.) tumor model ~2 × 10^6^ Panc-1-cells resuspended in 50 µl PBS were injected subcutaneously into the posterior region of the mouse trunk of female 5 to 6-week-old CD^®^nuBR mice (crl:CD1-Foxn1^nu^) Charles River Laboratories, Sulzfeld, Germany) through a 26-gauge needle. The injection sites were examined daily. Ten days post-injection the appearing tumors were measured twice weekly using a caliper and the volumes were calculated with the formula for ellipsoids (V = 4/3 π × (width/2)^2^ × (length/2)^2^.

For the orthotopic (ortho) tumor model tumor cell inoculation was performed by direct injection of tumor cells in the pancreatic tail of 5 to 6-week-old female CD^®^nuBR mice according to the procedure previously described in detail [32]. Briefly, a laparotomy was performed, the spleen with tail of the pancreas was exteriorized with cotton swabs, and with an insulin syringe 27G × 1.2 in., 1.0 ml (Terumo, Eschborn, Germany) approx. 2 × 10^6^ cells in 20 µl PBS of cell lines Panc-1 or Su.86.86 were gently injected into the pancreatic parenchyma. After completion of the surgical procedure, mice were inspected daily, the wound healing, body weight and physical condition of the animals was monitored over the total experimental time. Tumor growth was assessed by high-frequency ultrasound imaging, with a Vevo 770^®^ High-Resolution Imaging System, (Visual Sonics, Amsterdam, Netherlands).

The subcutaneous and orthotopic tumors were allowed to grow for about 4–5 weeks until approximately 0.5 cm^3^ in size. When the in vivo experiments were finished, animals were sacrificed and tumors and organs of interest were excised, divided into one part for flash- freezing in liquid N_2_ and storage at −80 °C for subsequent analyses and the other part for formalin-fixing and embedding in paraffin wax for histopathology analysis, IHC and Prussian blue staining.

### Magnetic resonance imaging

Relaxation measurements where performed using a 1.5 Tesla generally available human whole-body MR-scanner (Siemens Magnetom Symphony, Erlangen, Germany) with a radiofrequency coil/resonator optimized for animals for signal reception. The mouse coil was designed as a cylindrical volume resonator with an inner diameter of 35 mm and a usable length of 100 mm. For body temperature control the resonator was integrated in a spiral tube water jacket maintained at 37 °C.

For MRI examination and catheterization, tumor bearing female mice were anesthetized by inhalation of a mixture of isofluorane (1.5%), and O_2_. The flow was adjusted to the individual need of the animal. The tail vein was catheterized using a 30 G needle connected to a 10 cm PE 10 fine bore 0.28 mm ID; 0.61 mm OD polyethylene catheter (Smiths Medical International Ltd., Kent, UK) filled with 0.9% NaCl. Successful puncture of the tail vein was controlled by blood reflux into the catheter and by injection of 30 µl 0.9% NaCl. Subsequently the 1 ml syringe with 0.9% NaCl was replaced by a NPs containing syringe and the mouse with the tail-fixed catheter and syringe was placed into the animal resonator. Between 130 and 150 µl of NPs (10 mg/ml), respectively, were manually injected as bolus within 10–15 s into the tail vein of the nude mice.

All animals were examined with multiple axial high-resolution T2w turbo spin echo (TSE) pulse sequences, using the following imaging parameters: TR = 4390 ms, TE = 60 ms, TA = 7:38, NA = 5, field of view = 40 × 54 × 55 mm^2^, matrix = 144 × 192 × 46, slice thickness = 1.20 mm, voxel size = 0.28 × 0.28 × 1.20 mm^3^. During an overall measurement time of ~65 min, 8 measurements were obtained. Slices were placed to cover the liver, kidneys, muscle (the norm), the subcutaneous (sc) tumor, and the orthotopic (ot) tumor. The tissue types were analyzed in terms of the temporal behavior of the signal intensity.
